# Case Report: Uncommon MRI and PET/CT Imaging Findings in a Rare Case of Vertebral Sarcoidosis

**DOI:** 10.1155/crra/1056186

**Published:** 2026-11-05

**Authors:** Waqas Hassoon Hammadi Hammadi, Melpomeni Varvounis, Konstantina Foufa

**Affiliations:** ^1^ Department of Radiology Aarhus University Hospital Aarhus Denmark auh.dk; ^2^ Danish Hospital for Rheumatic Diseases Sønderborg Denmark; ^3^ Department of Radiology Lillebaelt Hospital Kolding Denmark sygehuslillebaelt.dk

**Keywords:** bone metastases, MRI, PET/CT, sclerotic pattern, vertebral sarcoidosis

## Abstract

Sarcoidosis is a systemic inflammatory granulomatous disease that can affect almost any body organ. Among the musculoskeletal manifestations, which are uncommon, vertebral sarcoidosis is extremely rare, while depicting a significant variability in radiological features. We present a unique case of a 52‐year‐old male, who was referred for MRI of the lumbar spine due to inflammatory back pain. Disc bulging at the intervertebral spaces L1/L2, L4/L5, and L5/S1 was found, without any signs of spinal stenosis or nerve root involvement. In addition, multiple small focal lesions were noticed in the vertebrae and the iliac bones, thus raising suspicion of bone metastases. Subsequent ^18^F‐FDG PET/CT showed neither evidence of primary tumor nor abnormal uptake in the vertebral lesions. No lytic or sclerotic pattern was recognized in CT scanning. Eventually, vertebral sarcoidosis was histologically confirmed by a nontargeted bone marrow biopsy of the iliac crest, performed as part of a standard hematological work‐up, which is the diagnostic gold standard for this pathologic entity. Follow‐up of the lesions with MRI scans showed stability over time, despite therapy with methotrexate.

## 1. Introduction/Background

Sarcoidosis is a multisystem inflammatory disease defined by the formation of noncaseating granulomas in affected organs [1]. The causes of sarcoidosis are still not clearly defined; the pathogenesis is considered though multifactorial. Research highlights that the disease is genetically, environmentally, and immunologically triggered [2], resulting in a dysregulated immune system forming granulomas and failing to resolve them. The annual incidence of sarcoidosis varies between 1 and 15 per 100,000 depending on race and geographic region [3]. Diagnosis is made possible by a combination of clinical, radiological and histological findings, and noncaseating granuloma is the hallmark of the disease [4].

The most common site of involvement is lungs and thoracic lymph nodes in > 90% of patients [3]. However, extrapulmonary manifestation can affect any body organ [5], and is observed in about 30% of the patients [3]. Musculoskeletal manifestations in sarcoidosis can present in the form of joint, bone, and muscle involvement [6]. Although joint involvement is relatively common (10%–35%), bone sarcoidosis is rather rare, with the frequency ranging from 3% to 13% [7]. It can affect any bone; the most commonly involved sites though are the hands and feet, followed by the long bones of the extremities [8]. Vertebral involvement is even rare and its radiologic features are nonspecific.

In this study, we present a biopsy‐verified case of vertebral sarcoidosis, with atypical magnetic resonance imaging (MRI) and ^18^F‐fludeoxyglucose positron emission tomography/computed tomography (PET/CT) findings, which overlap with a wide spectrum of pathological entities, primarily bone metastatic disease. This case report was prepared in accordance with the CARE (CAse REport) guidelines; a completed CARE checklist and a structured patient timeline are provided as Supporting Information.

## 2. Case Presentation

A 52‐year‐old male was referred from a general practitioner to our department for a lumbar MRI due to progressive lower back pain, fatigue, and morning stiffness, worsening over a 2‐year period. Our patient experienced minimal improvement by physiotherapy. There were no systemic symptoms, such as fever, weight loss, or night sweats, and physical examination did not reveal neurological deficits or significant joint abnormalities. No systemic diseases mentioned at the MRI referral.

An initial MRI of the lumbar spine in February 2023 showed disc bulging at the intervertebral spaces L1/L2, L4/L5, and L5/S1 without spinal stenosis or nerve root involvement. Multiple small focal lesions (most of which measuring < 1 cm) were noticed in the vertebral bodies, pedicles, and transverse and spinous processes, whereas a few larger lesions were seen in the iliac bones. These lesions exhibited high signal intensity in short tau inversion recovery (STIR) sequences and low signal intensity in T1‐ and T2‐weighted sequences, indicating sclerotic pattern (Figure 1). The differential diagnosis of small osseous lesions is quite wide including bone metastatic disease, hematopoietic diseases, atypical benign lesions (the commonest being atypical hemangiomas), infection, and granulomatous diseases.

**Figure 1:**
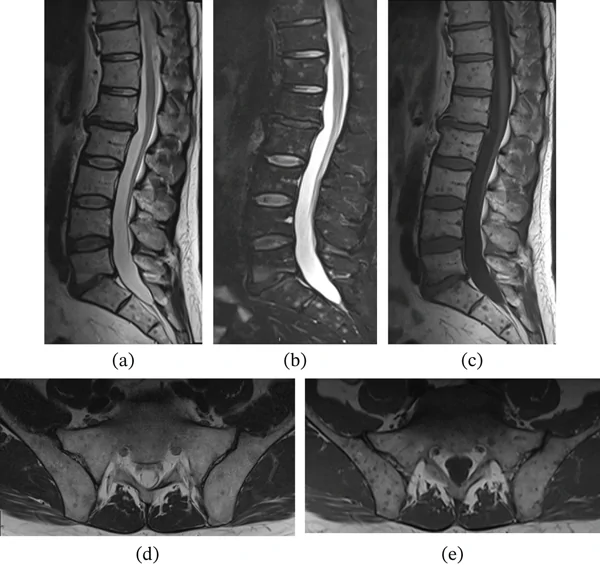
Vertebral sarcoidosis. Sagittal (a) T2, (b) STIR and (c) T1; axial (d) T2 and (e) T1 images demonstrate miliary T1‐ and T2‐hypointense and STIR‐hyperintense lesions in vertebral bodies and posterior elements as well as sacrum and iliac bones.

The original MRI protocol did not include intravenous contrast administration or diffusion‐weighted imaging sequences; we acknowledge this as a technical limitation of the initial diagnostic work‐up. On the available T1‐, T2‐ and STIR‐weighted sequences, the paravertebral soft tissues were unremarkable, with no soft tissue extension, and there was no evidence of epidural extension, cortical destruction, or vertebral height loss/fracture at the level of the sclerotic lesions.

Routine blood work, including prostate‐specific antigen (PSA) and myeloma protein (M‐component) tests, was normal. In our institution, a PET/CT scan with diagnostic, contrast enhanced CT component is performed in the majority of patients with suspicion of metastatic disease and unknown primary focus. PET/CT scanning did not show any signs of primary malignant lesions. Moreover, no increased FDG uptake was found corresponding to the MRI findings. Uptake was assessed qualitatively on visual review; quantitative SUVmax measurements were not generated for these lesions, in keeping with our department′s standard qualitative reporting practice for this indication, and no objective uptake values are therefore available for either PET/CT examination performed in this patient. Surprisingly there was not found a conspicuous morphologic CT equivalent to the vertebral lesions seen on MRI. Nevertheless, this scan showed bilateral hilar lymphadenopathy and perihilar opacities, thus raising suspicion of sarcoidosis (Figure 2).

**Figure 2:**
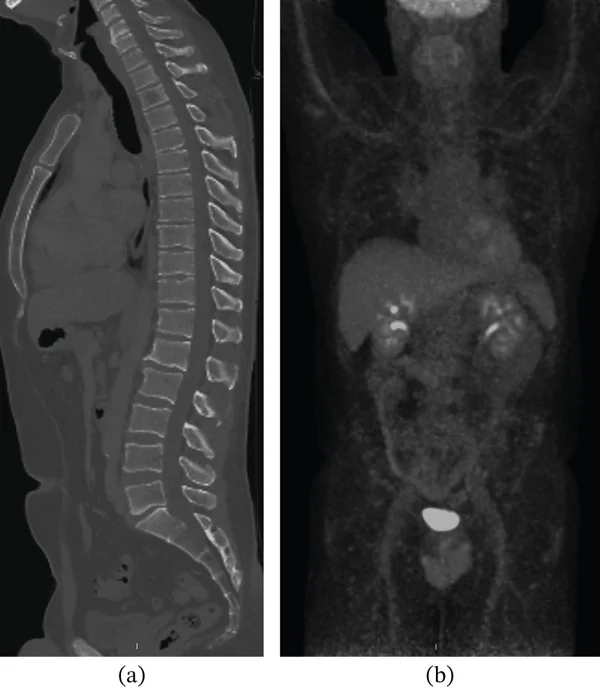
Vertebral sarcoidosis. ^18^F‐FDG PET/CT: (a) Sagittal CT‐bone and (b) MIP reconstructions show no morphological or metabolic correlate to MRI findings.

This scanning was subsequently presented to our pulmonologist conference, which revealed a history of pulmonary sarcoidosis in complete remission.

The patient′s pulmonary sarcoidosis had originally been diagnosed in August 2018, confirmed by endobronchial ultrasound‐guided transbronchial biopsy demonstrating nonnecrotizing granulomatous inflammation, with a mildly elevated serum angiotensin‐converting enzyme (ACE) of 89 U/L and normal ionized calcium (1.30 mmol/L) at diagnosis. This history had not been included in the initial referral for lumbar MRI, which had been requested outside the patient′s established pulmonology follow‐up after an occupational back injury; it was retrospectively identified at the multidisciplinary pulmonology conference. The patient had received oral prednisolone from 2020 to October 2023 during a period of exacerbated dyspnoea and declining lung function, which was gradually tapered and discontinued. Throughout the subsequent osseous work‐up, serum ACE fluctuated mildly (approximately 65–107 U/L) without marked elevation, and serial pulmonary function tests and chest CT remained essentially stable, indicating that the patient′s pulmonary sarcoidosis was clinically and radiologically quiescent during this period.

Since the clinical and laboratory work‐up has excluded a malignancy, the patient was referred to physiotherapy with minimal improvement. A year later due to persistent symptoms and clinical suspicion of spondyloarthritis, another MRI of the spine was performed and affirmed the presence of unchanged focal lesions in the lumbar spine. This repeat MRI examination likewise did not include intravenous contrast administration or diffusion‐weighted imaging sequences, consistent with the original imaging protocol. At that time, a nontargeted bone marrow biopsy of the iliac crest, performed as a standard hematological sampling procedure rather than a lesion‐directed biopsy, which revealed the presence of slightly hypocellular (for the patient′s age) bone marrow and a single nonnecrotizing granuloma, without giant cells or asteroid/Schaumann bodies documented on the original pathology report; dedicated microbiological special stains for acid‐fast bacilli and fungal organisms were not performed on the specimen, although serologic testing for tuberculosis and viral hepatitis was negative. These findings, together with exclusion of malignancy and infection and the patient′s known history of systemic sarcoidosis, suggested the diagnosis of sarcoidosis. Flow cytometric analysis of the same bone marrow specimen incidentally identified a low‐count monoclonal B cell lymphocytosis (MBL) with a chronic lymphocytic leukemia (CLL)–like immunophenotype, histologically and spatially distinct from the nonnecrotizing granuloma; karyotyping was not performed on this specimen. The hematology department classified this as an incidental finding of uncertain long‐term significance rather than an active hematological malignancy, and it was not considered to account for the vertebral imaging findings. This assessment was based on three considerations: First, the clonal B cell population was histologically and spatially separate from the nonnecrotizing granuloma within the same specimen, rather than colocalized with it; second, the clone size was very low (low‐count MBL), a burden generally considered insufficient to produce macroscopic, imaging‐detectable marrow lesions; and third, marrow infiltration by a lymphoproliferative clone typically produces a diffuse or homogeneous signal alteration rather than the multiple discrete, sclerotic‐pattern lesions observed on MRI in this patient.

Consequently, the patient was set on methotrexate therapy by April 2024, and follow‐up MRI and PET/CT scans, performed approximately 1 year after treatment initiation, showed persistent degenerative changes and no significant improvement in bone lesions. New‐onset dactylitis in the right third digit was observed, aligning with sarcoidosis‐related manifestations. The patient continued to experience back pain, improving with physical strain, as well as residual neck tension and morning stiffness lasting up to 30 minutes. Current management includes ongoing methotrexate therapy, physiotherapy targeting spinal mobility, and periodic imaging for monitoring of lung and osseous lesions.

## 3. Discussion

Sarcoidosis is a systemic inflammatory granulomatous disease that can involve any organ, resulting in a wide range of clinical features. In a review article of 22 cases of vertebral sarcoidosis, the lesions in all cases were diagnosed during a workup for back pain, which occurred also in our patient [9]. The lumbar pain was thought to be related to disc bulging at the intervertebral spaces L1/L2, L4/L5, and L5/S1 and not to vertebral lesions. In other cases, the lesions were discovered incidentally or due to complications such as neurological impairment or pathological fractures [10, 11].

Although involvement of the peripheral skeleton is not uncommon, vertebral involvement is thought to be rare despite a few published case reports. In the majority of the published data, the patients have a known history of lung sarcoidosis [12–14]. Similarly, our patient had a history of sarcoidosis, which unfortunately was not included in the initial MRI referral.

In a cohort of 48 patients with osseous sarcoidosis, vertebral sarcoidosis was found to have a male predominance [12]. The thoracic and the upper lumbar spine are more frequently affected, but multiple vertebral involvements are also possible. Any part of the vertebra can be affected, although vertebral body involvement was found to be more common compared with pedicles and posterior arches involvement [12, 15].

More recent data provide additional context regarding the frequency of axial skeletal involvement among patients with confirmed osseous sarcoidosis. A recent multicenter Italian cohort of 867 patients with sarcoidosis identified bone involvement in 5.3% of patients, with the pelvis and vertebrae representing the most frequently affected skeletal sites (54.3% and 52.2% of patients with bone involvement, respectively) [16]. Although osseous sarcoidosis overall remains uncommon, this suggests that vertebral involvement, once bone sarcoidosis is identified, may be relatively frequent, particularly with the increasing use of sensitive imaging modalities such as MRI and PET/CT.

To further substantiate the novelty of the imaging combination observed in our patient, Table 1 summarizes the imaging, histological, treatment and follow‐up features of previously published cases of vertebral sarcoidosis alongside the present case.

**Table 1:** Imaging, histological, treatment and follow‐up features of previously published cases of vertebral sarcoidosis and the present case.

Reference	Study type	MRI	CT	PET/CT (FDG)	Histology	Treatment	Follow‐up
Poyanli et al. [9]	Single case	Multiple vertebral lesions	Not detailed in available abstract	Not applicable (predates routine PET use)	Not detailed in available abstract	Not detailed	Not detailed
Sakellariou et al. [17]	Single case + literature review	Improved on follow‐up imaging	—	—	Consistent with sarcoidosis	Methotrexate monotherapy	Clinical improvement; normalized ACE/inflammatory markers; radiological improvement
Lefere et al. [18]	Single case, long‐term follow‐up	Multiple vertebral lesions; gradual fatty involution over 4 years	—	—	Not detailed in available abstract	Corticosteroids	Favorable long‐term outcome with fatty involution
Ben Hassine et al. [12]	Cohort, *n* = 48 (osseous sarcoidosis)	Vertebral body more often affected than posterior elements	—	—	—	—	Male sex associated with vertebral involvement
Yanny et al. [13]	Case series (axial osseous sarcoidosis)	—	Lytic/sclerotic patterns, pseudo‐Pott′s, “grid” appearance	—	—	—	—
Present case, 2026	Single case	Multiple small sclerotic‐pattern lesions (STIR‐hyperintense, T1/T2–hypointense); no contrast/DWI performed; paravertebral soft tissues unremarkable	No corresponding morphological correlate on CT	Completely negative FDG uptake despite multiple MRI lesions	Single small nonnecrotizing granuloma on nontargeted bone marrow biopsy (iliac crest); incidental low‐count MBL, CLL‐like	Methotrexate	Radiologically stable lesions without regression despite treatment

Conventional radiology does not play a significant role in the diagnosis of vertebral sarcoidosis. CT scanning is sensitive, yet lacks specificity. CT can reveal both lytic and sclerotic vertebral lesions, but also other features, like pseudo‐Pott′s disease and grid appearance [13]. It can also help guide bone biopsy [19, 20]. This was not the case in our patient, although a sclerotic pattern was expected according to the interpretation of MRI signal of the lesions. Surprisingly the lesions were not visible in CT scanning, which is one of the unique features in this case‐report.

MRI is thought to be the gold standard test for depicting lesions as well as detection of soft tissue and bone marrow extension [21]. Lytic lesions typically exhibit low‐signal intensity on T1‐weighted images and high‐signal intensity on T2‐weighted images with variable enhancement. In contrast, sclerotic lesions appear as low‐signal intensity on both T1‐ and T2‐weighted images, as in our case. Moreover, MRI is used in disease monitoring and assessment of therapy response. In previously published cases, complete resolution or replacement with fat or fibrous tissue was noticed after therapy with corticosteroids and/or methotrexate [17, 18]. This type of response was not encountered in our patient. Vertebral lesions were found to be unchanged 1 year after the initial diagnosis, possibly due to their fibrotic nature.

^18^F‐FDG PET/CT has emerged as a useful modality in systemic evaluation of sarcoidosis, since it was shown to be more sensitive for assessing the activity of the disease, monitoring treatment response, and detecting occult diagnostic biopsy sites [22]. Typically, bone lesions show increased FDG uptake [23]. In our patient, PET/CT scanning did not exhibit pathological uptake, which adds to the overall atypical radiological features. Several mechanisms may explain the absence of FDG uptake despite multiple MRI‐detectable lesions. The lesions may represent metabolically inactive or “burnt‐out” granulomas, in which active inflammatory infiltration has been replaced by fibrosis, reducing glucose avidity; alternatively, the MRI signal pattern itself may correspond to chronic, low‐turnover bone remodeling rather than active granulomatous inflammation, which is typically associated with higher FDG avidity. The overall low disease activity in our patient—whose pulmonary sarcoidosis was in clinical and radiological remission throughout the work‐up, with only mildly fluctuating ACE levels and normal pulmonary function—may also have been insufficient to generate a detectable metabolic signal. This interpretation is supported by recent evidence linking PET/CT metabolic parameters to biochemical markers of disease activity: Incekara et al. [24] demonstrated significant associations between total SUVmax and serum ACE levels, and between extrapulmonary metabolic activity and serum calcium levels, in patients with sarcoidosis, reinforcing that the PET‐negative appearance in our case likely reflects an exceptional, low‐activity disease state rather than a typical pattern, and underscoring the complementary rather than standalone role of FDG‐PET/CT in evaluating osseous sarcoidosis activity. As with the other modalities, PET/CT suffers from poor specificity and fails to distinguish sarcoidosis from other bone lesions. Therefore, histological sampling remains the gold standard for the definitive diagnosis of vertebral sarcoidosis; however, this distinction depends on whether the biopsy directly targets the suspected lesion or, as in our case, consists of nontargeted bone marrow sampling, which yields a lower pretest probability of lesion‐specific confirmation and relies more heavily on correlation with the overall clinical and imaging picture.

As it was highlighted earlier in the text, our case demonstrates some unique features among the other reported cases of vertebral sarcoidosis: miliary small lesions diffusely located in multiple vertebral bodies, also in the posterior compartments, MRI signal intensity indicating sclerotic lesions, yet no signs of sclerosis in CT scanning. In addition, absent FDG uptake in PET/CT and persistency of the lesions, despite methotrexate therapy. The principal educational message of this case is the marked discordance across imaging modalities in a single patient: multiple lesions clearly detectable on MRI, complete absence of a corresponding morphological abnormality on CT, and absence of pathological FDG uptake on PET/CT. This discordance underscores that a negative CT or PET/CT does not exclude vertebral sarcoidosis when MRI findings are suggestive, and reinforces the importance of considering this entity in the differential diagnosis of multiple vertebral lesions even when other modalities appear unremarkable. To our knowledge, there has not yet been reported a similar case of vertebral sarcoidosis with these imaging features. Our aim is to contribute to the acknowledgement of this entity and to point out its significance in the differential diagnoses of bone metastatic disease.

## Funding

No funding was received for this manuscript.

## Consent

Written informed consent was obtained from the patient for the publication of this case report and any accompanying images.

## Conflicts of Interest

The authors declare no conflicts of interest.

## Supporting Information

Additional supporting information can be found online in the Supporting Information section.

## Supporting information


**Supporting Information 1**  

**Supporting Information 2**  

## Data Availability

Data sharing is not applicable to this article as no datasets were generated or analyzed during the current study.
