# Supplementary material for: Case Report: Uncommon MRI and PET/CT Imaging Findings in a Rare Case of Vertebral Sarcoidosis
Source: Case Rep Radiol. 2026 Nov 5;2026:1056186. doi: 10.1155/crra/1056186 (PMC13740372; doi:10.1155/crra/1056186)
Supplement: Supplementary file 1 [file CRRA-2026-1056186-s001.docx]

**CARE Checklist**

*Manuscript 1056186 — Uncommon MRI and PET/CT Imaging Findings in a Rare Case of Vertebral Sarcoidosis*

| **#** | **Topic** | **Item** | **Reported (Y/N/NA)** | **Location in manuscript** |
| --- | --- | --- | --- | --- |
| 1 | Title | Identify the report as a case report and include the diagnosis/intervention of interest | Y | Title page |
| 2 | Keywords | 2-5 keywords identifying diagnoses/interventions | Y | After Abstract |
| 3 | Abstract | Introduction, case presentation, discussion, conclusion (structured or unstructured) | Y | Abstract |
| 4 | Introduction | Brief background referencing relevant medical literature | Y | Introduction/Background |
| 5 | Patient information | De-identified demographics; main concerns/symptoms; medical, family, psychosocial history relevant to case; relevant past interventions and outcomes | Y (demographics, presenting symptoms, relevant sarcoidosis history); family/psychosocial history not applicable | Case presentation |
| 6 | Clinical findings | Relevant physical examination findings | Y | Case presentation |
| 7 | Timeline | Historical and current information from this episode of care, organized as a timeline (figure/table) | Y | Supplementary Timeline (separate file) |
| 8 | Diagnostic assessment | Diagnostic methods, challenges, diagnostic reasoning, prognostic characteristics where applicable | Y (imaging, laboratory, histopathology, and clinical reasoning detailed) | Case presentation and Discussion |
| 9 | Therapeutic intervention | Types of intervention, administration, changes with rationale | Y (methotrexate, physiotherapy) | Case presentation |
| 10 | Follow-up and outcomes | Clinician/patient-assessed outcomes, important follow-up test results, intervention adherence/tolerability, adverse events | Y | Case presentation |
| 11 | Discussion | Strengths/limitations of the approach, relevant literature, rationale for conclusions, primary take-away lesson | Y | Discussion |
| 12 | Patient perspective | Patient's own perspective/experience where possible | N - not available/not collected; noted as a limitation | Not applicable |
| 13 | Informed consent | Did the patient give informed consent? State if not obtained/waived per journal policy | Y | Consent section |
