# Supplementary material for: Case Report: Uncommon MRI and PET/CT Imaging Findings in a Rare Case of Vertebral Sarcoidosis
Source: Case Rep Radiol. 2026 Nov 5;2026:1056186. doi: 10.1155/crra/1056186 (PMC13740372; doi:10.1155/crra/1056186)
Supplement: Supplementary file 2 [file CRRA-2026-1056186-s002.docx]

**Structured Patient Timeline**

*Manuscript 1056186 — Uncommon MRI and PET/CT Imaging Findings in a Rare Case of Vertebral Sarcoidosis (CARE guideline supplementary item)*

| **Date** | **Event** |
| --- | --- |
| Aug 2018 | Chest X-ray and CT thorax suspicious for sarcoidosis; EBUS-TBNA (22 Aug) confirmed non-necrotizing granulomatous inflammation (30 Aug); serum ACE 89 U/L, ionized calcium 1.30 mmol/L; pulmonary function normal |
| 2020 – Oct 2023 | Oral prednisolone for progressive dyspnoea/decline in lung function, gradually tapered and discontinued |
| Autumn 2022 | Occupational back injury (lifting furniture); progressive low back pain thereafter |
| 28 Feb 2023 | Lumbar spine MRI (no IV contrast, no DWI): numerous small (<1 cm) foci, low T1/T2 and high STIR signal, raising suspicion of a neoplastic (sclerotic) process; paravertebral soft tissues unremarkable |
| 6 Mar 2023 | 18F-FDG PET/CT (whole-body, tumour protocol): no increased FDG uptake in the skeleton; known pulmonary/hilar changes unchanged |
| 20 Mar 2023 | Multidisciplinary pulmonology conference: findings judged unchanged since 2018 and attributed to known sarcoidosis |
| 4 Apr 2023 – 25 Sep 2023 | Serum ACE fluctuating mildly (normal to slightly elevated); pulmonary function and chest CT stable |
| 31 Jan 2024 | Referral to rheumatology for persistent back pain; ACE 71 U/L |
| 7 Feb 2024 | Repeat whole-spine MRI (spondyloarthropathy protocol): lesions unchanged; findings considered more compatible with bone sarcoidosis than metastasis |
| 13 Mar 2024 | Multidisciplinary radiology conference: recommended repeat PET/CT and bone marrow biopsy |
| 18 Mar 2024 | Bone marrow biopsy of the iliac crest (standard, non-targeted haematological sampling procedure): slightly hypocellular marrow for age; single small non-necrotizing granuloma; incidental low-count monoclonal B-cell lymphocytosis (MBL, CLL-like immunophenotype) on flow cytometry |
| 21 Mar 2024 | Repeat 18F-FDG PET/CT: no increased uptake in bone marrow; unchanged overall |
| 12 Apr 2024 | Pulmonary sarcoidosis considered in remission; osseous involvement diagnosed; methotrexate initiated (up-titrated to 20 mg weekly) with folic acid; annual MRI follow-up planned |
| 5 Sep 2024 | New dactylitis of the right third digit; ultrasound-confirmed grade III flexor tenosynovitis, treated with local corticosteroid injection |
